# Physics-Guided Machine Learning for Performance Prediction and Multi-Objective Optimization of High-Conductivity Aluminum Conductors

**DOI:** 10.3390/ma19091839

**Published:** 2026-04-29

**Authors:** Yaojun Miao, Zhikang Cao, Tong Yao, Yufei Wang, Haiyan Gao, Jun Wang, Baode Sun

**Affiliations:** Shanghai Key Lab of Advanced High-Temperature Materials and Precision Forming and State Key Lab of Metal Matrix Composites, School of Materials Science and Engineering, Shanghai Jiao Tong University, Shanghai 200240, China; miaoyaojun@ztt.cn (Y.M.);

**Keywords:** aluminum conductor, Equivalent Solute–Heat Index, physics-guided machine learning, multi-objective optimization

## Abstract

Producing high-conductivity aluminum conductors for power transmission involves 23 trace elements and multiple interconnected thermo-mechanical stages. The ultra-low alloying levels required to preserve high electrical conductivity create a narrow compositional window and highly imbalanced distributions, which hinder traditional data-driven learning. Here, we developed a physics-guided machine-learning framework based on 4458 valid industrial production records to predict tensile strength and electrical resistivity. In addition to raw composition and process parameters, we introduce ratio descriptors (e.g., Fe/Si and Al/Si) and propose a physics-informed metric termed the Equivalent Solute–Heat Index (ESHI) to couple key solute chemistry (Si, Fe, B) with normalized thermal-history intensity. Fe and Si primarily influence resistivity through impurity/solute scattering, while B mainly affects microstructural uniformity via grain refinement. Incorporating ESHI as an augmented signal into the best-performing XGB surrogate markedly improves generalizability, increasing the tensile strength R^2^ from 0.75 to ~0.92. SHAP analysis reveals that ESHI dominates the decision logic by modulating both targets with metallurgically interpretable mechanisms: solute-controlled scattering and thermal history-traced second-phase evolution that stabilizes the microstructure. NSGA-III was further employed to map the Pareto front and identify composition–process combinations that optimize the strength–conductivity trade-off, enabling improved mechanical reliability while minimizing resistive losses in practical power-transmission applications. Experimental validation on industrial wires confirms this reliability.

## 1. Introduction

High-conductivity aluminum conductors are widely used in overhead transmission lines and modern renewable power systems due to their low density, cost efficiency, and excellent electrical conductivity [1]. In practical service conditions, conductor materials must not only maintain high electrical conductivity but also possess sufficient mechanical strength to ensure long-term operational reliability [2]. Therefore, achieving precise strength regulation while maintaining minimal conductivity degradation has become a critical challenge in the manufacturing of high-conductivity aluminum wires.

Unlike conventional multi-component strengthening aluminum alloys, industrial high-conductivity aluminum conductors are characterized by extremely low microalloying contents. To preserve electrical conductivity, elements such as Si, Fe, and B are typically restricted to trace or sub-per-mille levels, while the addition of other strengthening elements is also strictly limited. As a result, the compositional adjustment window of such systems is extremely narrow, and the distribution of process variables is highly concentrated [3]. Under these conditions, property variations often originate from the combined effects of minute compositional fluctuations and subtle thermal-history perturbations. Consequently, the effective signal contained in the data is inherently weak, whereas industrial production noise, measurement uncertainty, and process fluctuations are unavoidable, forming a typical low-signal–high-noise modeling environment [4].

In recent years, machine learning techniques have shown significant potential in predicting material properties, particularly in addressing complex nonlinear relationships among multiple variables [5]. However, most existing studies focus on alloy systems with relatively wide compositional ranges or clearly distinguishable strengthening mechanisms [6,7]. For systems with extremely narrow compositional windows, such as high-conductivity aluminum conductors, conventional machine learning models often struggle to identify stable patterns within highly concentrated variable distributions [8]. As a result, models may achieve reasonable fitting performance on training datasets but exhibit limited generalization capability when applied to unseen production conditions [9].

One important reason for this limitation is that most machine learning models treat raw compositional variables and process parameters as independent input features [10,11]. Within a narrow compositional range, however, the statistical variance of these variables is extremely limited, making it difficult for purely data-driven algorithms to amplify meaningful signals. In reality, even slight variations in trace solute elements may produce nonlinearly amplified effects under specific thermal histories, influencing mechanisms such as solute stability, impurity scattering, second-phase evolution, and microstructural freezing. If these composition–thermal history interactions are not explicitly represented in the feature space, the model may fail to capture the underlying physical trends hidden in low-amplitude data [12].

To address this challenge, physics-guided machine learning provides a promising strategy for industrial process modeling [13,14]. Instead of reconstructing complex metallurgical processes through fully physical models, this approach introduces descriptors with explicit physical meaning to enhance the model’s sensitivity to key mechanisms [15]. In narrow-window alloy systems, such descriptors can effectively amplify physically relevant signals without significantly increasing model complexity, thereby improving the ability of machine learning models to distinguish subtle property variations. In the high-conductivity aluminum conductor system, Si and Fe act as dominant impurity elements controlling electron scattering and second-phase formation, while trace B plays an important role in grain refinement and structural uniformity [16]. Meanwhile, thermal parameters such as melt temperature and exit temperature after rolling strongly influence solute distribution and microstructural stabilization. Based on these considerations, coupling key trace elements with an equivalent thermal-history intensity may provide a physically meaningful descriptor for capturing the dominant metallurgical mechanisms governing property variations [17].

Therefore, this study proposes a physics-guided feature termed the Equivalent Solute–Heat Index (ESHI) to characterize the coupling effects between trace solute elements and normalized thermal history. Based on 9800 industrial production records collected from a continuous manufacturing line, 4458 valid samples were obtained after data cleaning and anomaly removal. Machine learning models were then developed to predict tensile strength and electrical resistivity, and the influence of the proposed physics-guided descriptor on model generalization was systematically evaluated. The objective of this work is to establish a transferable feature-engineering framework for narrow compositional window alloy systems, providing both theoretical insights and practical guidance for composition–process co-optimization and intelligent manufacturing of aluminum conductors.

## 2. Data Source and Variable Definition

### 2.1. Industrial Source and Dataset Scale

The dataset used in this study was collected from a continuous industrial production line for high-conductivity aluminum conductors. The alloy system is based on industrially pure aluminum, where mechanical strength and microstructural stability are regulated through extremely low levels of microalloying elements while maintaining stringent electrical conductivity requirements. The production process involves several key stages, including melt preparation, continuous casting, hot rolling, and online property inspection, representing a typical large-scale continuous manufacturing scenario.

**Figure 1:**
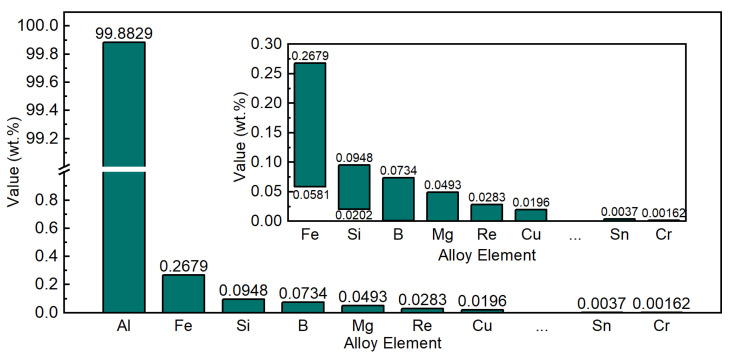
Distribution of major compositional variables in the industrial high-conductivity aluminum conductor dataset.

Unlike conventional multi-component strengthening aluminum alloys, the compositional design of high-conductivity aluminum conductors is strictly constrained by electrical conductivity requirements. Aluminum accounts for approximately 99.88 wt.% of the material, while most solute elements exist at trace or sub-per-mille levels. The total alloying content is generally below 1.3 wt.%, and the allowable compositional fluctuation range is extremely limited. As illustrated in Figure 1, the distribution of compositional variables is therefore highly concentrated across production batches.

Under such conditions, statistical differences among batches are relatively small, whereas variations in mechanical and electrical properties arise primarily from the combined influence of subtle compositional fluctuations and thermal-history perturbations during processing. This results in a characteristic low-variance yet strongly coupled data structure, in which the effective signal contained in the compositional and process variables is relatively weak compared with unavoidable industrial noise and measurement uncertainty. Such characteristics make the construction of robust predictive models particularly challenging.

The original database contained approximately 9800 independent production records, each corresponding to a complete manufacturing cycle and associated with measured tensile strength and electrical resistivity values. To ensure data reliability, systematic data cleaning and consistency checks were performed on the raw dataset. After removing incomplete entries, abnormal records, and statistically inconsistent samples, 4458 valid samples were retained for model development. In addition, 447 samples were reserved as a completely independent test set to evaluate the generalization capability of the trained models under unseen production conditions.

### 2.2. Feature Set Curation

In narrow compositional window industrial systems, the organization of the feature space plays a crucial role in determining the effectiveness of machine learning models. In this study, a multi-level variable system was constructed, including composition variables, thermo-mechanical process variables, and performance response variables.

(1) Composition variables

The compositional variables are based on industrially pure aluminum and mainly focus on trace elements that influence electrical conductivity and microstructural stability. The dataset includes 23 compositional variables in total. Among them, Si and Fe are the dominant impurity elements. Although their concentrations are extremely low, they significantly affect electron scattering behavior and second-phase formation. B acts as a trace grain-refining element and plays an important role in regulating grain size and microstructural uniformity. In addition, several microalloying elements such as Mg, Ti, and Zr are also included.

It should be noted that the compositional fluctuation range of these elements is extremely narrow [18]. For most key elements, the variation amplitude is typically below the sub-per-mille level, as illustrated in Figure 1. From a statistical perspective, most compositional variables exhibit highly concentrated distributions, with standard deviations much smaller than their mean values. Such characteristics imply that single compositional variables alone rarely show strong linear correlations with material properties.

(2) Thermo-mechanical process variables

In addition to composition variables, the dataset systematically records 16 process parameters associated with the continuous manufacturing process. These variables describe the thermal and deformation history experienced by the material during production. The casting stage includes parameters such as melt temperature, casting speed, and cooling-related conditions, while the rolling stage records inlet temperature, exit temperature, rolling mill current, and wire speed.

In high-conductivity aluminum systems, where compositional adjustment is highly constrained, thermal history often acts as a key factor that amplifies subtle compositional variations. For example, melt superheat can influence solute distribution homogeneity, while the exit temperature after rolling determines microstructural stabilization during rapid solidification and deformation. Although the numerical fluctuation ranges of these process parameters are also relatively limited due to production stability requirements, they may exhibit nonlinear coupling effects with trace solute elements [19].

(3) Performance response variables

Two key properties were selected as prediction targets in this study: tensile strength and electrical resistivity. Tensile strength reflects the combined influence of microstructural characteristics and dislocation structures, with a typical variation range of approximately 110–150 MPa in the dataset. Electrical resistivity is primarily governed by solute atom scattering and impurity distribution homogeneity, with a relatively narrow fluctuation range of 27.3–27.9 nΩ·m.

Compared with tensile strength, the variation range of resistivity is significantly smaller, indicating that the response variable itself also exhibits a highly concentrated distribution. This low-amplitude response characteristic further increases the difficulty of constructing reliable predictive models.

(4) Derived features and physics-guided descriptors

To enhance the structural representation of interactions among variables, several derived compositional features were constructed based on the original dataset. For example, elemental ratios such as Fe/Si were introduced to reflect the potential tendency for second-phase formation and impurity interactions.

However, within a narrow compositional window, individual variables or simple ratios may still provide limited discriminatory power. Therefore, a physics-guided descriptor, termed the Equivalent Solute–Heat Index (ESHI), was further proposed. This descriptor couples key trace solute concentrations with a normalized thermal-history intensity, enabling a structured integration of composition and process information. By amplifying the physically meaningful coupling effects between trace elements and thermal conditions, the ESHI descriptor improves the model’s ability to identify subtle composition–process–property relationships hidden in low-variance industrial data. The mathematical formulation and construction procedure of the ESHI descriptor will be presented in detail in the following section.

### 2.3. Data Cleaning and Statistical Analysis

**Figure 2:**
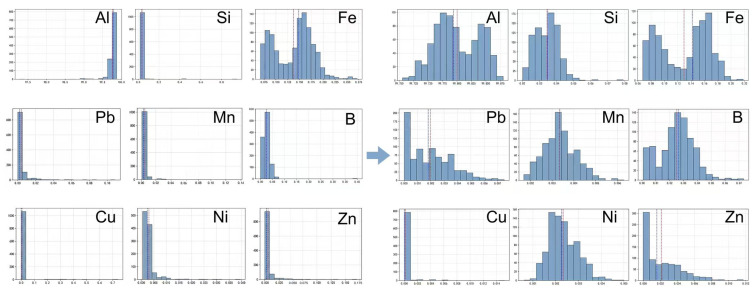
Distribution of representative compositional variables after data cleaning.

Before model construction, the original dataset containing approximately 9800 production records was subjected to outlier detection and physical consistency verification. Abnormal data were identified through a combination of statistical screening and engineering threshold evaluation to remove erroneous measurements while preserving genuine industrial fluctuations. After data cleaning, 4458 valid samples were retained for model training. Figure 2 shows the distribution of representative compositional variables after cleaning. Most elements exhibit highly concentrated distributions within narrow compositional ranges, reflecting the strict conductivity constraints of high-conductivity aluminum conductors. Compared with conventional alloy datasets, the overall variance of the present dataset is significantly smaller.

In Figure 2, several elements (e.g., Al, Si, Fe, Pb, Mn, B, and Ni) exhibit sharp and high-intensity peaks in their histograms. These “intense signals” do not indicate measurement artifacts or abnormal outliers; rather, they reflect the intrinsic characteristics of industrial conductor-grade aluminum production, where both chemistry and process are tightly controlled. Specifically, (i) the permissible composition window is extremely narrow to meet stringent conductivity specifications, so most batches cluster around fixed setpoints; and (ii) industrial feeding strategies and analytical resolution can further discretize the recorded values, producing concentrated bins and pronounced peaks. As a result, the marginal variance of many single-element features is very small, and the property variation is not driven by wide changes in individual elemental contents.

Importantly, the concentrated distributions imply that tensile strength and electrical resistivity variations mainly originate from coupled effects: minute compositional fluctuations interacting with thermal history and microstructural evolution, rather than from any single element showing a broad range. Therefore, the peaked histograms in Figure 2 are a quantitative manifestation of the “low-variance, weak-signal” learning environment. This observation motivates the use of derived descriptors (e.g., elemental ratios) and, more importantly, physics-guided coupled features such as ESHI, which explicitly encode composition–thermal history interactions and thus amplify physically meaningful signals for model learning and generalization.

## 3. Method

### 3.1. Overall Modeling Framework

To address the challenges of extremely small compositional fluctuations, narrow property ranges, and weak statistical signals in high-conductivity aluminum conductors, a physics-guided machine learning framework was developed to simultaneously predict tensile strength and electrical resistivity.

**Figure 3:**
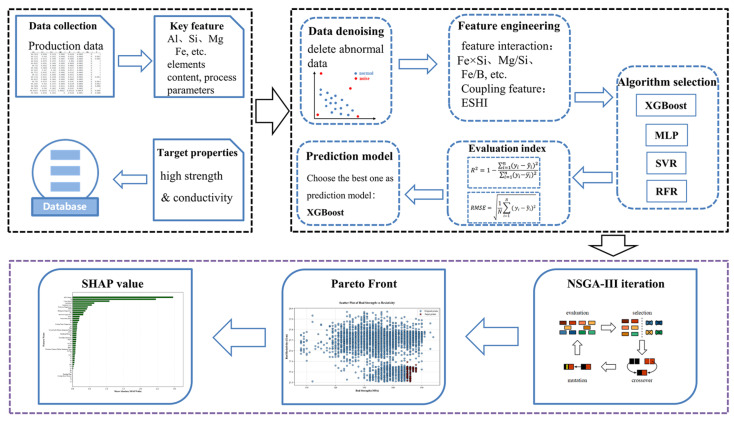
Schematic illustration of the physics-guided machine learning framework used in this study.

The overall workflow is illustrated in Figure 3, which consists of four main stages: industrial data preprocessing, physics-guided feature construction, model training and validation, and model interpretability analysis.

In conventional industrial data modeling, all compositional and process parameters are typically treated as independent input variables for model training. However, in high-conductivity aluminum systems, most variables exhibit highly concentrated distributions within narrow ranges, making it difficult for individual variables to show clear statistical correlations with performance.

To address this limitation, the present study introduces physically meaningful coupled descriptors to enhance the structural representation of composition–process interactions. These physics-guided features are incorporated into the model together with the original variables, enabling the model to better capture subtle property variations without significantly increasing model complexity.

### 3.2. Feature Engineering

In high-conductivity industrial aluminum systems, both compositional and process variables are distributed within highly concentrated and narrow ranges, making it difficult for individual variables to exhibit clear statistical correlations with material properties. To enhance the model’s ability to capture underlying physical relationships, composite features and physics-guided coupled descriptors were constructed while retaining the original variables.

First, several ratio-type compositional features were introduced to characterize the relative relationships among elements. Because the absolute concentration variation in each element is extremely limited, the relative ratios between elements may more sensitively reflect tendencies of second-phase formation and solute competition behavior. Accordingly, several proportional descriptors were constructed, including $c_{\mathrm{Fe}}/c_{\mathrm{Si}}$, ${c_{\mathrm{Al}}/c}_{\mathrm{Si}}$ and cZr/cRe. These composite features do not introduce additional physical assumptions but instead reorganize the original variables to enhance the distinguishability of subtle compositional variations in statistical space [20]. Such representations enable machine-learning models to more effectively capture potential nonlinear interactions among compositional variables [21].

For physics-guided feature construction, the evolution of properties in high-conductivity aluminum conductors is mainly governed by trace solute elements and their response under thermal history conditions. Based on this consideration, an Equivalent Solute–Heat Index (ESHI) was proposed. Within the solute subspace, Si, Fe, and B were identified as key controlling elements. Silicon mainly influences electron scattering and solid-solution behavior; iron has extremely low solubility in aluminum and tends to form second-phase particles that affect microstructural uniformity; boron plays a critical role in grain refinement and microstructural scale regulation. These three elements correspond respectively to atomic-scale scattering mechanisms, phase-structure evolution mechanisms, and microstructural regulation mechanisms, forming a minimal closed control subspace from a physical perspective.

To avoid potential physical distortion introduced by simple equal-weight superposition and to allow different mechanisms to contribute differently to the combined descriptor, weighting coefficients $\omega_{1}$, $\omega_{2}$ and $\omega_{3}$, were introduced. The weighted solute term is defined as [20]:

(1) $$C_{solute}={\omega_{1}c}_{Si}+{\omega_{2}c}_{Fe}+\omega_{3}c_{B}$$

where $c_{i}$ represents the mass fraction (wt.%) of the corresponding element, the weighting coefficients describe the relative contributions of different solute mechanisms in the combined descriptor. Although the concentration levels of these elements are similar in magnitude under the narrow compositional window, their physical effects on electron scattering, second-phase formation, and microstructural regulation are not equivalent. Introducing weighting factors, therefore, improves the physical rationality of the solute descriptor while maintaining a simple mathematical form. To ensure reproducibility and stability, the weight parameters were optimized during model training through cross-validation rather than predetermined empirical values. Considering that complete temperature–time evolution curves are difficult to obtain in continuous industrial production, a dimensionless equivalent thermal history term was introduced:

(2) $$g(\mathrm{process})=\frac{T_{\mathrm{furnace}}-T_{\mathrm{exit}}}{T_{\mathrm{furnace}}}$$

where $T_{\mathrm{furnace}}\;$ represents the furnace temperature and $T_{\mathrm{exit}}$ denotes the rod exit temperature after rolling. This expression reflects the relative proportion of thermal energy released during the forming process and can be regarded as a compressed representation of the driving force for microstructure freezing and solute redistribution.

Finally, the Equivalent Solute–Heat Index is defined as:

(3) $$\mathrm{ESHI}=\left(\omega_{1}c_{\mathrm{Si}}\;|\;\omega_{2}c_{\mathrm{Fe}}\;|\;\omega_{3}c_{B}\right)\cdot \frac{T_{\mathrm{furnace}}-T_{\mathrm{exit}}}{T_{\mathrm{furnace}}}$$

The ESHI descriptor is incorporated together with all original compositional and process variables as model inputs. By explicitly coupling solute concentration with thermal history intensity, this descriptor enhances the representation of composition–process interactions in feature space, thereby improving model generalization performance when applied to narrowly distributed industrial datasets.

### 3.3. Algorithmic Selection and Performance Evaluation

After feature construction, several regression algorithms were employed to establish property prediction models, including Extreme Gradient Boosting (XGB), Random Forest (RF), Support Vector Regression (SVR), and Multilayer Perceptron (MLP). All models were trained using the same dataset partition and feature system to ensure a fair comparison among different algorithms.

During training, five-fold cross-validation was adopted to optimize model hyperparameters. The dataset was repeatedly divided into training and validation subsets to reduce the dependence of model performance on a particular data split and to improve the stability of parameter selection. After hyperparameter optimization, the final model performance was evaluated using a completely independent test set, which reflects the generalization capability of the model under previously unseen production conditions.

Model performance was evaluated using two metrics [22]: the coefficient of determination ($R^{2}$) and the root mean square error (RMSE). The coefficient of determination is defined as:

(4) $$R^{2}=1-\frac{\sum_{i=1}^{N}(y_{i}-{\hat{y}}_{i}{)}^{2}}{\sum_{i=1}^{N}(y_{i}-\overset{ˉ}{y}{)}^{2}}$$

where $y_{i}$ and ${\hat{y}}_{i}$ denote the measured and predicted values, respectively, and $\overset{ˉ}{y}$ represents the mean value of the observations. The $R^{2}$ metric describes the proportion of variance in the target variable explained by the model.

The root mean square error is defined as:

(5) $$RMSE=\sqrt{\frac{1}{N}\sum_{i=1}^{N}(y_{i}-{\hat{y}}_{i}{)}^{2}}$$

which directly quantifies the average magnitude of prediction errors and has the same physical unit as the target property. For high-conductivity aluminum conductors, where the property variation range is relatively narrow, the combined use of $R^{2}$ and RMSE provides a more reliable evaluation of predictive performance than relying on a single metric.

In addition, SHapley Additive exPlanations (SHAP) were employed after model training to analyze the contribution of individual features to model predictions. Particular attention was paid to the changes in feature importance before and after introducing the ESHI descriptor, in order to evaluate the effectiveness of the physics-guided feature in improving model interpretability and generalization performance.

## 4. Results and Discussion

### 4.1. Modeling Process of ML Surrogates

To systematically evaluate the applicability of different algorithms to industrial datasets with narrow compositional windows, four representative regression models were selected for comparison: Extreme Gradient Boosting (XGB), Random Forest (RF), Support Vector Regression (SVR), and Multilayer Perceptron (MLP). These models represent four typical nonlinear modeling frameworks, including gradient boosting tree ensembles, bagging-based ensembles, kernel-based regression methods, and neural networks, respectively. Such a selection enables a comprehensive comparison of different machine learning paradigms in the context of industrial metallurgical data [15,19,23].

Random Forest is a classical ensemble tree model that generally exhibits good robustness to noise and nonlinear relationships. However, when variables are distributed within highly concentrated ranges, and feature redundancy is high, the splitting strategy may be influenced by weak statistical signals, which can limit extrapolation performance [24]. SVR maps the input space into a high-dimensional feature space through kernel functions and theoretically possesses strong nonlinear approximation capability. Nevertheless, its performance is sensitive to feature scaling and hyperparameter selection, particularly in high-dimensional datasets with weak signal intensity [25]. MLP, as a feed-forward neural network model, provides strong nonlinear modeling capacity but typically requires larger datasets and careful regularization strategies to avoid overfitting [26]. For industrial datasets with narrow variable ranges and relatively low signal-to-noise ratios, model stability can therefore become a challenge. In comparison, XGB, based on the gradient boosting decision tree framework, constructs residual learners sequentially and incorporates explicit regularization to control model complexity [27]. This approach can effectively capture high-order feature interactions while maintaining tolerance to multicollinearity among variables. Consequently, XGB has demonstrated strong predictive capability and reasonable interpretability in many industrial materials prediction tasks [26,27,28].

In this study, the modeling task was formulated as a supervised regression problem with two continuous target variables: tensile strength and electrical resistivity. Considering the differences in their underlying physical mechanisms and statistical distributions, two independent regression models were constructed to avoid potential interference between targets and to facilitate subsequent interpretability analysis. After data cleaning, 4458 samples were used for model training, while 447 samples were reserved as a completely independent test set. The test set was strictly excluded from model training and hyperparameter optimization and was only used for the final evaluation of model generalization performance. To ensure fair comparison among algorithms, all models were trained using the same feature system, including original compositional variables, process variables, and constructed composite descriptors. Because different algorithms exhibit different sensitivities to feature scaling, continuous variables were standardized before training the SVR and MLP models. For tree-based models, both original and normalized feature spaces were examined to verify their robustness to feature scaling.

It should be noted that industrial continuous production data inevitably contain residual noise and process fluctuations originating from raw material variation, measurement uncertainty, and production rhythm changes. Such noise cannot be eliminated through simple statistical preprocessing. Therefore, in addition to average prediction accuracy, model robustness and generalization ability were regarded as critical evaluation criteria. A model exhibiting large fluctuations during cross-validation, even with a relatively high average $R^{2}$, would not be considered suitable for practical industrial deployment.

### 4.2. Hyperparameter Optimization and Cross-Validation Results

To ensure that model performance reflects the intrinsic learning capability rather than accidental parameter initialization, all models were optimized using grid search combined with five-fold cross-validation on the training dataset. This strategy balances parameter-space coverage and computational efficiency, enabling the identification of stable and reproducible optimal model configurations.

The hyperparameter optimization strategies for different algorithms are summarized as follows:

Tree-based models (XGB and Random Forest): key parameters including tree depth, number of estimators, learning rate, subsampling ratio (subsample), and column sampling ratio were tuned to control model complexity and reduce overfitting; SVR: the kernel type, penalty coefficient (C), and kernel parameter (γ) were optimized to balance margin maximization and error tolerance; MLP: network depth, number of neurons, activation functions, and L2 regularization strength were adjusted, combined with an early-stopping strategy to improve training stability.

Model performance was evaluated using the coefficient of determination ($R^{2}$) and root mean square error (RMSE). In addition, performance variations among the five validation folds were examined to assess model sensitivity to data partitioning and potential overfitting. Table 1 summarizes the prediction performance of different models for tensile strength and electrical resistivity using the feature system consisting of original compositional and process variables.

For tensile strength prediction, XGB achieved the highest $R^{2}$ value of 0.75 and the lowest RMSE of 3.96, outperforming the other models. For electrical resistivity prediction, SVR obtained the highest $R^{2}$ (0.78) with the lowest RMSE (0.07). However, its performance on tensile strength prediction was relatively poor, indicating limited overall predictive capability across multiple targets.

In contrast, XGB exhibited consistently strong performance for both properties, achieving a $R^{2}$ of 0.75 for resistivity prediction while maintaining the best accuracy for strength prediction. Random Forest showed moderate performance for tensile strength ${\;R}^{2}$ = 0.66 but performed poorly for resistivity prediction $R^{2}$ = 0.31, suggesting that the bagging-based splitting mechanism may struggle to extract dominant interaction structures when variables are distributed within narrow ranges. The MLP model also showed relatively limited performance ($R^{2}$ ≈ 0.58 for strength prediction), indicating that neural networks may suffer from training instability when applied to datasets with limited sample size and low signal amplitude.

**Figure 4:**
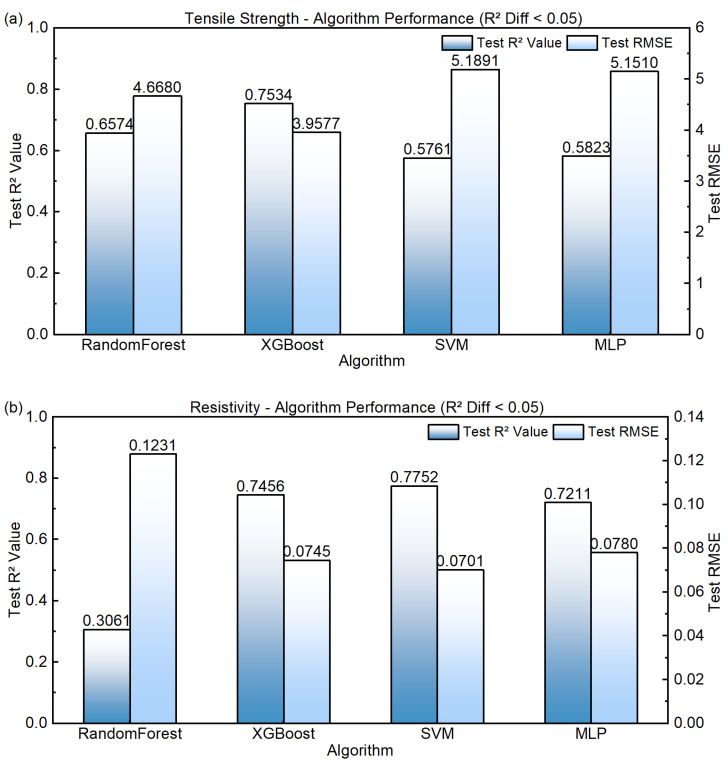
Comparison of generalization performance among the four machine learning models. Panel (**a**) shows the generalization ability of the strength model, and Panel (**b**) shows that of the resistivity model.

The comparison of model generalization performance is further illustrated in Figure 4, which presents the predictive results of the four algorithms on the independent test set.

The observed performance differences can be largely attributed to the statistical characteristics of the industrial dataset. In high-conductivity aluminum conductor production data, the compositional variables exhibit extremely low concentrations and narrow fluctuation ranges due to strict conductivity constraints. Meanwhile, process parameters are relatively concentrated but contain multi-scale fluctuations. Under such conditions, linear correlations between composition and process variables are generally weak, and higher-order nonlinear interactions mainly govern property variations. In this “weak-signal, strong-nonlinearity, and narrow-distribution” data environment, XGB effectively captures complex feature interactions through gradient-boosting residual learning and hierarchical feature splitting, while explicit regularization helps control model complexity. As a result, XGB demonstrates superior predictive accuracy, cross-validation stability, and test-set generalization capability.

Based on these results, XGB was selected as the primary model for subsequent analysis, including the evaluation of physics-guided features and mechanistic interpretation.

### 4.3. Performance Enhancement by Physics-Guided Feature (ESHI)

After determining XGB as the baseline model, the Equivalent Solute–Heat History Index (ESHI) was introduced to enhance the representation of composition–thermal history coupling mechanisms.

**Figure 5:**
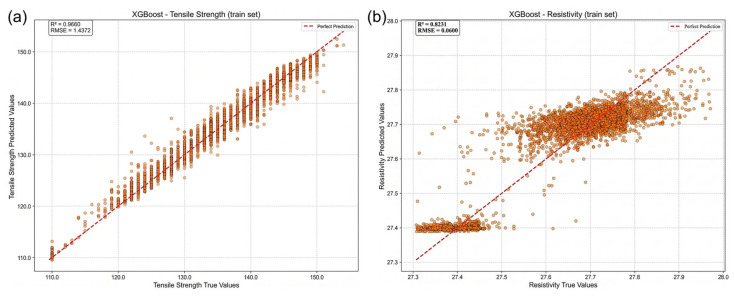
Influence of the ESHI feature on model predictive performance: (**a**) tensile strength prediction; (**b**) electrical resistivity prediction.

As an initial evaluation, the weighting coefficients *ω*_1_, *ω*_2_ and *ω*_3_ were assigned equal values ($\omega_{1}{:\omega }_{2}:\omega_{3}$ = 1:1:1). Under this condition, the impact of the physics-guided feature on model performance was examined. Within the XGB framework, the introduction of ESHI resulted in a significant improvement in predictive accuracy. For tensile strength prediction, the coefficient of determination increased from 0.753 to 0.905, while the RMSE decreased from 3.958 to 2.404. For electrical resistivity prediction, $R^{2}$ increased from 0.746 to 0.830, and RMSE decreased from 0.075 to 0.060. The corresponding results are presented in Figure 5.

These results demonstrate that even without distinguishing the relative contributions of different solute mechanisms, the simple construction of a composition–thermal history coupling descriptor can significantly improve model performance. This indicates that in industrial datasets with narrow compositional windows, treating original variables as independent inputs is insufficient to capture underlying mechanism coupling, whereas physics-guided compressed descriptors can enhance structural representation in the feature space.

However, from the perspective of physical mechanisms, equal weighting provides only mathematical symmetry rather than mechanistic symmetry. In high-conductivity aluminum conductor systems, the roles of Si, Fe, and B in property evolution differ significantly. Si and Fe mainly influence electron scattering and second-phase formation, thereby directly affecting resistivity and microstructural uniformity. In contrast, B primarily regulates microstructure through grain refinement and heterogeneous nucleation, operating at a different structural scale. Under conditions where microalloying concentrations are extremely low and compositional fluctuations are tightly constrained by conductivity requirements, the marginal sensitivity of each mechanism to property variation cannot be assumed to be identical.

Furthermore, when the amplitude of variable fluctuations is very small, equal weighting may partially mask the dominant mechanism signals within statistical noise, thereby limiting the discriminative capability of the constructed descriptor. Introducing differentiated weights can therefore strengthen the representation of key mechanisms in the feature space. To investigate the influence of weight allocation, a constraint condition was introduced during weight optimization: $\omega_{1}{+\omega }_{2}+\omega_{3}$ = 10. This constraint serves three purposes. First, fixing the total weight ensures that different weight combinations represent only relative contribution differences, avoiding numerical shifts caused by overall scaling. Second, because solute concentrations in high-conductivity aluminum systems are extremely small, moderately amplifying the overall magnitude of the weighted solute term allows its numerical range to become comparable to that of major process variables. This improves the sensitivity of tree-based models to feature splitting while maintaining numerical stability. Third, transforming the optimization problem into a ratio optimization problem reduces the parameter search space and improves interpretability.

**Figure 6:**
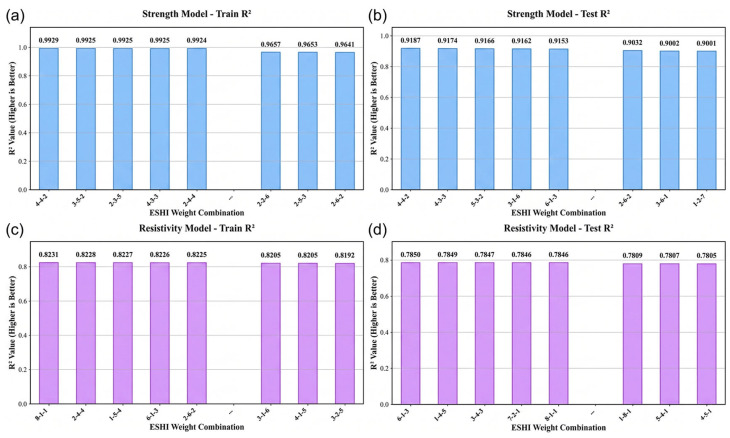
Influence of different weighting coefficients in ESHI on model generalization performance: (**a**) training ${\;R}^{2}$ of tensile strength models; (**b**) testing $\;R^{2}$ of tensile strength models; (**c**) training $R^{2}$ of resistivity models; (**d**) testing $R^{2}$ of resistivity models.

Under this constraint, systematic searches over different weight ratios were conducted. The results indicate that the optimal configurations for the two prediction tasks are different. When the weight ratio is $\omega_{1}{:\omega }_{2}:\omega_{3}$= 4:4:2, the tensile strength model achieves the best performance with an $R^{2}$ value of 0.917. For electrical resistivity prediction, the optimal performance is obtained at $\omega_{1}{:\omega }_{2}:\omega_{3}$= 6:1:3, where the model reaches an $R^{2}$ value of 0.831. The corresponding trends are illustrated in Figure 6.

These results indicate that the influence of weight allocation is more pronounced for tensile strength prediction. Because the microalloying concentrations are extremely low, property variations are not directly driven by absolute concentration changes but rather by differences in the relative sensitivity of underlying mechanisms. Weight optimization effectively enhances the representation of dominant mechanisms within the feature space, thereby improving model generalization capability under weak-signal conditions.

### 4.4. Independent Test Set Prediction and Generalization Verification

To further evaluate the true generalization capability of the proposed model, the optimized XGB–ESHI model was applied to the independent test dataset, and the predicted results were compared with the experimentally measured values of tensile strength and electrical resistivity. Since the test dataset was completely excluded from model training and hyperparameter optimization, the prediction results provide an objective assessment of the model’s extrapolation capability on unseen samples.

**Figure 7:**
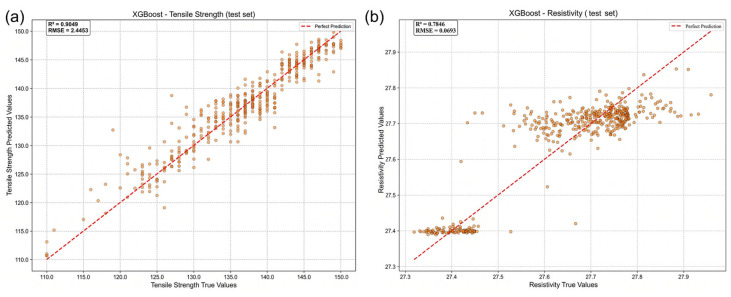
Comparison between predicted and measured values on the independent test dataset: (**a**) tensile strength prediction; (**b**) electrical resistivity prediction.

As shown in Figure 7, the tensile strength prediction on the test set achieved a $R^{2}$ value of 0.905 with an RMSE of 2.445, while the resistivity prediction obtained a $R^{2}$ value of 0.784 with an RMSE of 0.070. Although the performance is slightly lower than that observed during cross-validation on the training dataset, the overall accuracy remains high, and no significant performance collapse or structural deviation is observed.

From the distribution of predicted versus measured values, the ESHI-enhanced model demonstrates good consistency across the entire property range. Most data points are distributed close to the diagonal line, indicating the absence of systematic overestimation or underestimation. Notably, the model maintains stable prediction performance in the high-strength and low-resistivity regions, which correspond to the most critical performance ranges for industrial conductor applications.

Compared with the baseline model constructed using only the original compositional and process variables, the ESHI-enhanced model exhibits a clear improvement in predictive accuracy on the test set, with higher $R^{2}$ values and lower prediction errors. Importantly, this improvement is not achieved by increasing the number of features but rather by introducing a physics-guided compressed descriptor that embeds metallurgical mechanisms into the input feature space.

From a learning perspective, the ESHI descriptor effectively constrains the model from relying on accidental statistical correlations and instead encourages the learning of stable composition–thermal history–property coupling relationships. In this sense, ESHI functions as a form of implicit physical regularization: although it does not explicitly modify the loss function, it introduces mechanistic constraints into the feature space through structured representation, thereby improving model identifiability and stability in narrow-window industrial datasets.

### 4.5. Feature Importance and SHAP Interpretability Analysis

To further reveal the internal decision mechanisms of the model and verify its consistency with metallurgical principles, SHapley Additive exPlanations (SHAP) were employed to interpret the trained XGB–ESHI model. SHAP is based on game theory and quantifies the marginal contribution of each feature to individual predictions, enabling both global and local interpretability of machine learning models.

In the present analysis, the weight ratios corresponding to the best-performing models on the test set were adopted. The optimal ESHI weight ratios were $\omega_{1}{:\omega }_{2}:\omega_{3}$ = 4:4:2 for the tensile strength model and $\omega_{1}{:\omega }_{2}:\omega_{3}=$ 6:1:3 for the resistivity model.

(1) Feature contribution analysis for tensile strength

**Figure 8:**
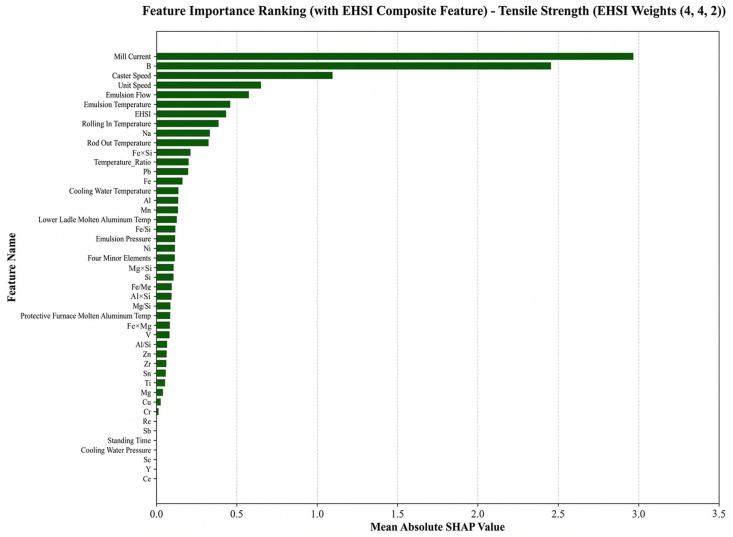
SHAP feature importance ranking for the tensile strength prediction model.

The SHAP results indicate that the most influential features for tensile strength prediction are rolling mill current, B content, rolling speed, emulsion flow rate, emulsion temperature, and ESHI, as shown in Figure 8. Several cooling-related parameters, including emulsion flow rate and emulsion temperature, rank among the most important variables. These parameters directly influence the cooling rate during processing and thus affect microstructural uniformity and solute distribution. The high importance of boron content is also consistent with its well-known role in grain refinement and heterogeneous nucleation control. The ranking of these features indicates that the model captures both microalloying-induced grain refinement mechanisms and deformation strengthening mechanisms. Therefore, tensile strength prediction is governed by the combined influence of composition and thermo-mechanical processing, reflecting the coupled evolution of microstructure under industrial processing conditions.

(2) Feature contribution analysis for electrical resistivity

**Figure 9:**
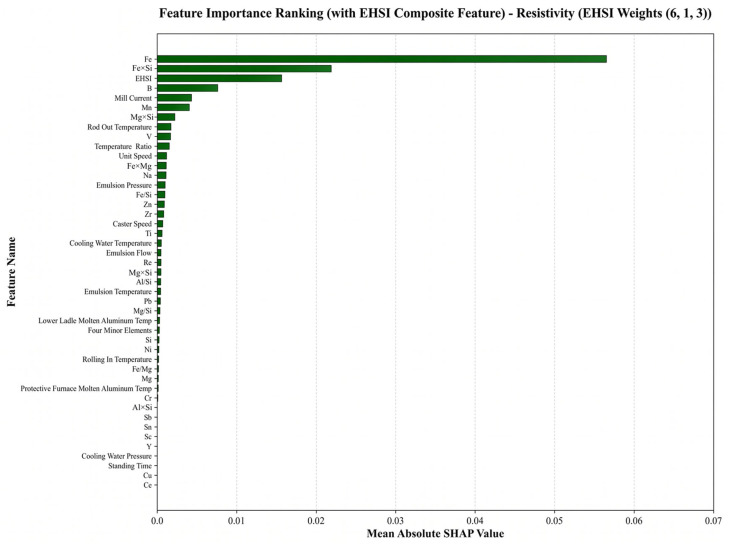
SHAP feature importance ranking for the electrical resistivity prediction model.

For electrical resistivity prediction, the most influential features include Fe content, Fe/Si ratio, ESHI, B content, and rolling mill current, as illustrated in Figure 9. Among these variables, Fe content and the Fe/Si ratio are strongly associated with the formation and distribution of second-phase particles, which can significantly affect electron scattering behavior. The prominence of compositional variables indicates that resistivity is highly sensitive to trace solute distribution. Even minor compositional fluctuations can amplify electron scattering effects if local compositional inhomogeneities or second-phase variations occur. The importance of Si content and Fe/Si ratio further highlights the critical role of second-phase formation behavior in determining electrical conductivity. In addition, process parameters such as holding time can influence solute redistribution and microstructural stability, thereby indirectly affecting resistivity.

(3) Physical consistency and mechanism verification

Importantly, the SHAP feature rankings exhibit strong consistency with established metallurgical theories. The key variables automatically identified by the model are closely associated with grain refinement mechanisms, second-phase regulation, and thermo-mechanical coupling during processing. This result indicates that the model does not rely on accidental statistical correlations but instead captures physically meaningful composition–process–property relationships. Notably, ESHI maintains a relatively high contribution in the global feature importance ranking, confirming its effectiveness in representing the coupled effects of solute concentration and thermal history. Combined with the previously observed improvements in prediction accuracy, these results demonstrate that the physics-guided descriptor not only enhances predictive performance but also improves the model’s ability to identify dominant metallurgical mechanisms. Therefore, the XGB–ESHI framework provides not only high prediction accuracy but also physically meaningful interpretability. This capability offers a promising approach for knowledge discovery and process optimization in industrial aluminum conductor production.

## 5. Multi-Objective Optimization and Experimental Validation

### 5.1. NSGA-III-Mapped Pareto Optimization

In this study, the NSGA-III (Non-dominated Sorting Genetic Algorithm III) was employed as the core multi-objective optimization method to identify optimal combinations of alloy composition and processing parameters. The optimization procedure was performed based on two pre-trained high-accuracy surrogate models constructed using XGB, which predict tensile strength and electrical resistivity of aluminum alloy rods. Before optimization, the feasible ranges of all input variables were defined according to metallurgical knowledge and industrial process constraints. These constraints included the upper and lower bounds of continuous variables as well as selectable sets for discrete parameters. In addition, compositional variables were subjected to an equality constraint requiring their total sum to be 100%, ensuring physical consistency within the search space.

**Figure 10:**
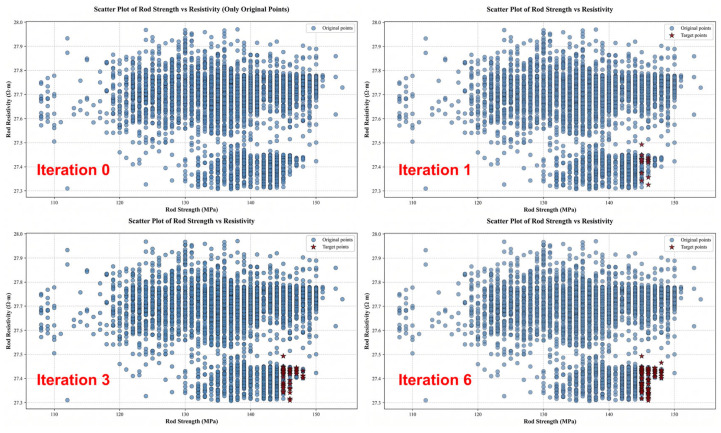
Pareto front obtained through NSGA-III multi-objective optimization.

During each evolutionary iteration, candidate solutions consisting of composition and process parameter combinations were evaluated using the trained surrogate models. The predicted tensile strength and resistivity values served as objective functions, replacing time-consuming physical experiments and enabling rapid evaluation of large numbers of candidate solutions. Through the evolutionary operations of selection, crossover, and mutation, combined with reference-point guidance and non-dominated sorting, the NSGA-III algorithm progressively explored the high-dimensional design space and converged toward the Pareto optimal front. After six optimization iterations with 15 candidate solutions per generation, a set of uniformly distributed Pareto-optimal solutions was obtained. These solutions are represented by the dark red star markers in Figure 10. Each Pareto solution corresponds to a feasible combination of alloy composition and processing parameters that achieves an optimal trade-off between the two conflicting objectives: maximizing tensile strength and minimizing electrical resistivity. These optimized solutions provide a set of promising candidates for subsequent experimental validation and customized alloy design.

### 5.2. Experimental Validation

**Figure 11:**
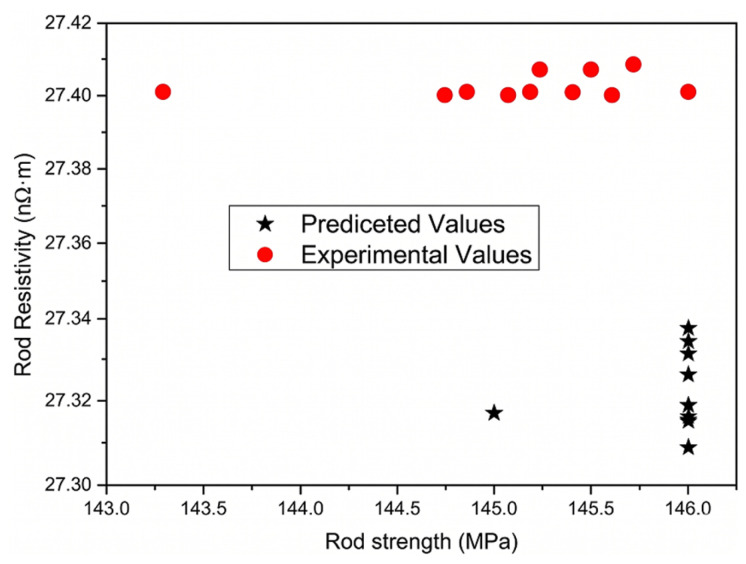
Comparison between predicted and experimentally measured properties of the optimized alloys.

To validate the reliability of the proposed optimization framework, experimental verification was performed based on the predicted Pareto solutions. From the optimized candidate set, ten solutions with tensile strength not lower than 145 MPa and the lowest predicted resistivity values were selected for experimental preparation using the corresponding compositions and process parameters suggested by the machine-learning optimization. The experimental results show that the measured tensile strength values are 1.3–1.4 MPa lower than the predicted values, while the measured electrical resistivity values are 0.06–0.08 higher than the model predictions, as illustrated in Figure 11. Despite these deviations, the differences remain within the RMSE prediction error range of the trained models. More importantly, the experimentally obtained properties are significantly superior to those observed in the original dataset, demonstrating that the machine-learning-assisted optimization successfully identifies high-performance parameter combinations.

These results confirm that the proposed XGB–ESHI–NSGA-III framework can effectively guide the discovery of improved alloy compositions and processing conditions. The approach enables efficient exploration of the composition–process design space and provides a reliable strategy for the data-driven development of high-strength, high-conductivity aluminum conductors.

## 6. Conclusions

This study developed and validated a physics-guided machine-learning framework to model and optimize the coupled composition–process–property relationships in industrial high-conductivity aluminum conductors under an ultra-narrow compositional window. The main conclusions are:

(1) From ~9800 industrial production records, 4458 valid samples were obtained after data cleaning, with an additional 447 samples reserved as a fully independent test set. The highly concentrated distributions of both trace-element chemistry and process variables confirm a typical low-variance, weak-signal learning environment for conductor-grade aluminum alloys.
(2) Among the evaluated regressors, XGB provided the best overall accuracy and stability across both targets. Introducing the proposed ESHI, which couples key solute chemistry (Si, Fe, B) with a normalized thermal-history intensity, substantially improved generalization. In particular, the tensile strength prediction improved from R^2^ ≈ 0.75 (baseline XGB) to ∼0.92 with ESHI, together with a clear reduction in prediction error (RMSE).
(3) SHAP analysis shows that the model decision logic is dominated by physically meaningful factors, including ESHI and key thermo-mechanical variables, and identifies composition-related terms (e.g., Fe content and Fe/Si ratio) as major drivers for resistivity. These rankings are consistent with metallurgical understanding of impurity/solute scattering, second-phase-related evolution, and microstructure stabilization in conductor alloys, indicating that the model captures stable mechanism-aligned patterns rather than accidental correlations.
(4) By integrating the trained XGB–ESHI surrogates with NSGA-III, a Pareto front balancing high tensile strength and low electrical resistivity was obtained, enabling practical selection of composition–process settings for specific conductor requirements. Industrial-wire experiments on selected Pareto candidates showed excellent agreement with model predictions, with deviations remaining within the RMSE level of the surrogates, demonstrating that the framework can reliably guide composition–process co-optimization in manufacturing.

## Figures and Tables

**Table 1:** Prediction performance of different models under the “composition + process parameter” feature combinations.

Model	Tensile Strength		Electrical Resistivity	
	R^2^	RMSE	R^2^	RMSE
RF	0.66	4.67	0.31	0.12
XGB	0.75	3.96	0.75	0.08
SVM	0.58	5.19	0.78	0.07
MLP	0.58	5.15	0.72	0.08

## Data Availability

The original contributions presented in this study are included in the article. Further inquiries can be directed to the corresponding authors.
